# A case of successful contribution of therapeutic drug monitoring of valganciclovir as the prophylaxis against cytomegalovirus infection in a lung transplant recipient

**DOI:** 10.1186/s40780-024-00352-y

**Published:** 2024-06-07

**Authors:** Yoshiki Katada, Keisuke Umemura, Shunsaku Nakagawa, Yurie Katsube, Masahiro Tsuda, Satona Tanaka, Hiroshi Date, Miki Nagao, Tomohiro Terada

**Affiliations:** 1 Department of Clinical Pharmacology and Therapeutics, Kyoto University Hospital, 54 Shogoin- Kawahara-Cho, Sakyo-Ku, Kyoto, 606-8507 Japan; 2 Department of Infection Control and Prevention, Kyoto University Hospital, 54 Shogoin- Kawahara-Cho, Sakyo-Ku, Kyoto, 606-8507 Japan; 3 Graduate School of Pharmaceutical Sciences, Kyoto University, 46-29 Yoshida-Shimo-Adachi-Cho, Sakyo-Ku, Kyoto, 606-8501 Japan; 4 Department of Thoracic Surgery, Graduate School of Medicine, Kyoto University, 54 Shogoin- Kawahara-Cho, Sakyo-Ku, Kyoto, 606-8507 Japan; 5 Department of Clinical Laboratory Medicine, Graduate School of Medicine, Kyoto University, 54 Shogoin- Kawahara-Cho, Sakyo-Ku, Kyoto, 606-8507 Japan

**Keywords:** Valganciclovir, Prophylaxis, Cytomegalovirus, Lung transplantation, Therapeutic drug monitoring

## Abstract

**Background:**

Ganciclovir and its prodrug, valganciclovir, are first-line agents for cytomegalovirus infection prophylaxis after lung transplantation. Although valganciclovir prophylaxis is known to result in severe leukopenia as an adverse effect, dosage adjustment based on therapeutic drug monitoring (TDM) of ganciclovir concentration is not generally implemented in clinical practice.

**Case presentation:**

In this report, we describe the case of a female in her fifties after lung transplantation who successfully maintained valganciclovir prophylaxis under TDM with a minimal occurrence of severe leukopenia. Valganciclovir administration was initiated at a conventional dose of 450 mg/day on postoperative day 43 but was reduced to 450 mg/2 days on postoperative day 69 because of a decrease in white blood cell count and an increase in trough ganciclovir concentration. Subsequently, the valganciclovir dose adjustment was switched from label-indicated renal function-guided dosing to TDM-based dosing, targeting a trough level of 300–800 ng/mL. This target range was determined through deliberations with infectious disease specialists and pharmacists based on previously reported data. The TDM-based dose adjustment successfully prevented cytomegalovirus reactivation without causing significant adverse effects. Valganciclovir prophylaxis was completed on postoperative day 256, and the patient was transferred to another hospital for rehabilitation.

**Conclusions:**

The findings of the present case suggest that TDM-based dosing could be helpful for clinicians in optimizing the prophylactic administration of valganciclovir in patients undergoing lung transplantation.

## Background

Cytomegalovirus (CMV) infection is the most common opportunistic infection among patients undergoing lung transplantation. CMV can cause severe symptoms such as retinitis, pneumonia, and colitis. In addition, CMV infection modulates host immune function, increasing the risk of rejection, invasive *Aspergillus* infections, and other opportunistic infections [1, 2]. Valganciclovir, an orally administered ganciclovir prodrug, is widely prescribed to prevent CMV infection after solid organ transplantations. Valganciclovir dosing is typically determined by renal function as its active metabolite, ganciclovir, is eliminated through the kidneys. Severe leukopenia may still manifest despite adjusting the dosage of valganciclovir according to renal function. Among lung transplant recipients receiving prophylactic valganciclovir, 25.5% (28 of 110) reportedly discontinued treatment due to adverse effects [3].

Therapeutic drug monitoring (TDM) is useful for early detection of adverse events and optimizing drug dosage. Although routine TDM of ganciclovir is not recommended in the international consensus guidelines [4], previous studies have suggested therapeutic and toxic concentration ranges for ganciclovir [5, 6]. Reports indicate that ganciclovir trough concentrations ranging from 1,000 to 2,000 ng/mL may effectively treat CMV infection. Conversely, subtherapeutic concentrations elevate the risk of CMV resistance and breakthrough viremia [7, 8]. Nevertheless, there are no established guidelines for optimal serum ganciclovir levels for prophylaxis of CMV infection. Although the threshold trough ganciclovir concentration associated with toxicity has not been fully elucidated, our group previously reported that a trough ganciclovir concentration of ≥ 872 ng/mL resulted in grade 3 or higher leukopenia in lung transplant recipients receiving valganciclovir prophylaxis [9]. Despite these findings, TDM of ganciclovir has not yet been implemented in clinical practice.

Here, we report the case of a lung transplant patient in whom the valganciclovir dose was adjusted based on TDM. In this patient, TDM-based dosing of valganciclovir enabled successful CMV prophylaxis without causing severe leukopenia.

## Case presentation

**Fig. 1:**
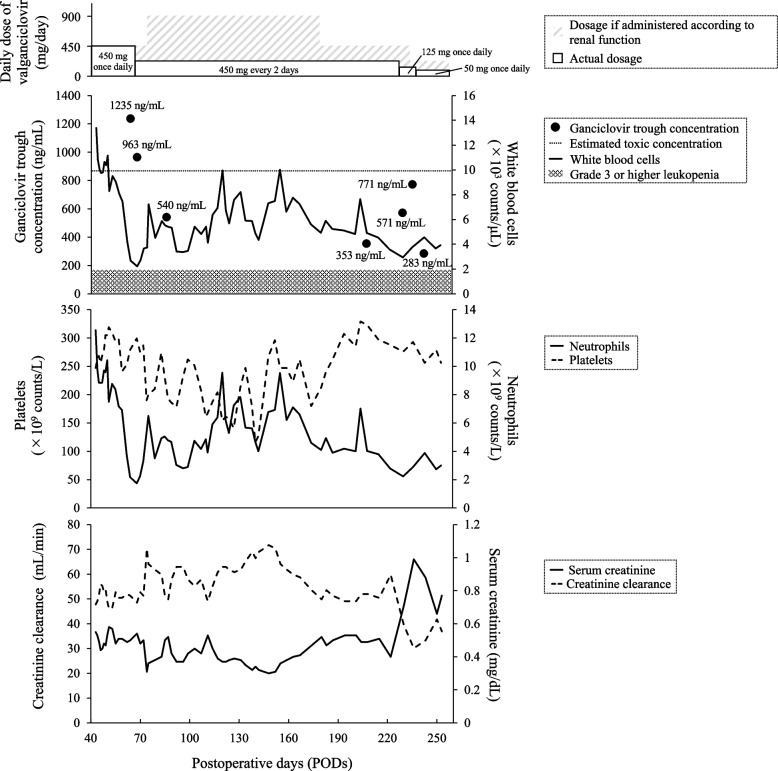
Clinical course post lung transplantation. Valganciclovir doses, laboratory data, and ganciclovir trough concentrations are shown. The white box indicates the actual dosage administered, whereas the shaded box represents the dosage based on renal function, as recommended by the instructions provided in the package insert. The lower detection limit for ganciclovir was 100 ng/mL. The upper limit of the ganciclovir trough concentration was 872 ng/mL, which is borderline for severe leukopenia

A woman in her fifties (height = 152 cm, weight = 37.5 kg, and body mass index = 16.2 kg/m^2^) underwent living-donor lobar lung transplantation to address thoracic sarcoidosis, pulmonary hypertension, and pulmonary aspergilloma. Both the recipient and donor tested positive for CMV IgG antibodies. Figure 1 shows the clinical course of valganciclovir doses, ganciclovir trough concentrations, and various other laboratory parameters. The patient was markedly cachexic at the time of transplantation. Consequently, postoperative recovery was slow, and the patient required a prolonged hospital stay.

The lung transplantation procedure was successful despite encountering excessive bleeding exceeding 11 L owing to severe pleural adhesion. Immunosuppressive regimens (basiliximab, mycophenolate mofetil, and corticosteroids) were initiated on postoperative day (POD) 1. Mycophenolate mofetil was orally administered at 1,000 mg/day, and the dosage was reduced in the presence of leukopenia. Corticosteroid therapy was initiated with intravenous methylprednisolone at 125 mg/day for 3 days after transplantation and reduced to a stable dose of oral prednisolone (0.4 mg/kg) using a weekly weaning regimen. Basiliximab was administered on PODs 1 and 5, followed by tacrolimus on POD 8, with trough concentrations maintained at 8–10 ng/mL. Antiviral prophylaxis with intravenous ganciclovir (2.5 mg/kg/day, as label-indicated) was initiated on POD 7 but temporarily discontinued on POD 11 due to the decrease in creatinine clearance (19 mL/min). Continuous hemodiafiltration was performed on PODs 12–21. The decrease in creatinine clearance was caused by acute right-sided heart failure associated with pulmonary artery stenosis. Subsequently, with pulmonary artery stenting, the hemodynamics improved, and creatinine clearance increased to 51 mL/min. During hospitalization, the patient was monitored weekly for CMV infection through microbiological blood tests for CMV (CMV antigen test, HRP-C7; LSI Medience, Tokyo, Japan). CMV reactivation was defined as the presence of more than 5/50,000 positive cells. The number of positive cells increased slightly to 3/53,200 on POD 43. Valganciclovir (450 mg/day; label-indicated dose) was initiated on POD 43. Moreover, antibody-mediated rejection developed on POD 74, necessitating therapeutic intervention involving plasma exchange from POD 74 to 78, a steroid pulse on POD 109 to 111, and anti-thymocyte globulin from POD 119 to 125.

The patient experienced continuous post-hemodiafiltration withdrawal and exhibited relatively impaired renal function, leading us to hypothesize that this condition might delay ganciclovir excretion and heighten the risk of severe leukopenia. To avoid cessation of valganciclovir prophylaxis, we performed TDM to maintain trough concentrations of ganciclovir below 872 ng/mL, which is less toxic than that previously reported by our group [9]. Serum ganciclovir concentrations were measured using liquid chromatography-tandem mass spectrometry as described previously [9]. The lower limit of quantification of ganciclovir using this method was 100 ng/mL. For this case, the target trough concentration range for ganciclovir was established at 300–800 ng/mL following consultations with infectious disease specialists and pharmacists to mitigate the risk of toxicity. Although the prophylactic target concentration range of GCV has not been established, it was anticipated, based on our previous study [9], that maintaining the trough blood concentration of GCV above 300 ng/mL would prevent CMV reactivation. The trough concentration of ganciclovir was 1,235 ng/mL on POD 64 and 963 ng/mL on POD 68. The white blood cell count decreased from 4,190/μL (POD 62) to 2,220/μL (POD 68) and was considered an adverse reaction to valganciclovir. Therefore, the valganciclovir dose was reduced from 450 mg/day to 450 mg/2 days on POD 69. The mycophenolate mofetil dose was reduced from 1,000 to 500 mg/day. Owing to the presence of melena and anemia, an upper gastrointestinal endoscopy was performed on POD 89, which revealed multiple gastric ulcers. A real-time polymerase chain reaction performed on a sample taken from the patient’s stomach was negative for CMV, ruling out a gastrointestinal CMV infection. The patient was prescribed vonoprazan for the management of multiple gastric ulcers. On POD 85, the white blood cell count improved to 5,520/μL, and the ganciclovir trough concentration decreased to 540 ng/mL. On POD 230, the white blood cell count had decreased to 2,950/μL. The trough concentration of ganciclovir showed an upward trend, reaching 571 ng/mL. Meanwhile, the serum creatinine increased from 0.4 mg/dL (POD 222) to 0.71 mg/dL (POD 230), and the creatinine clearance decreased from 59.8 mL/min (POD 222) to 39.4 mL/min (POD 230). Therefore, the valganciclovir dose was reduced from 450 mg/ 2 days to 125 mg/day (dry syrup formulation) on POD 231. On POD 236, the white blood cell count improved to 3,790/μL, but renal function worsened with a creatinine level of 0.99 mg/dL and a creatinine clearance of 30.1 mL/min. Additionally, because the ganciclovir trough concentration was 771 ng/mL, nearing the upper limit of the toxic range, the dosage of valganciclovir was reduced from 125 mg/day to 50 mg/day (dry syrup formulation). On POD 237, a gradual decrease in renal function was observed, and the patient was diagnosed with calcineurin inhibitor nephropathy and vitamin D-related toxicities. As a result, eldecalcitol 0.75 µg was discontinued because it was challenging to reduce the dose of tacrolimus from the standpoint of rejection prevention. After the discontinuation of eldecalcitol, renal function improved. On POD 243, the white blood cell count improved to 4,550/μL, and the ganciclovir trough concentration decreased to 283 ng/mL. On POD 256, the prophylactic administration of valganciclovir for CMV was completed because the risk of CMV infection was low. The patient was transferred to another hospital for rehabilitation on POD 257. During the observation period, we adjusted the ganciclovir trough concentration to within the optimal range of 300–800 ng/mL and noted no incidence of severe leukopenia related to valganciclovir administration. Furthermore, neutropenia and thrombocytopenia were not severe (Fig. 1), and no other adverse events or CMV reactivation were observed.

## Discussion and conclusions

In this case, the initial valganciclovir dosage was established considering renal function; nevertheless, the patient encountered a decline in the white blood cell count to 2,220/μL. The results of our previous study suggested that a ganciclovir trough concentration of 872 ng/mL or higher is associated with severe leukopenia [9]. We adjusted the valganciclovir dosage based on the trough serum concentrations of ganciclovir and successfully administered it without CMV reactivation or significant adverse effects. The TDM of ganciclovir has been suggested to be effective in preventing severe leukopenia in lung transplant patients receiving valganciclovir prophylaxis.

Patients eligible for lung transplantation often present with sarcopenia as a result of chronic obstructive pulmonary disease and chronic respiratory failure, which results in low muscle mass [10]. Consequently, this can cause low serum creatinine levels, which, in turn, leads to an overestimation of both creatinine clearance and estimated glomerular filtration rate (eGFR) when calculated based on serum creatinine levels [11]. Therefore, in lung transplant recipients with lower muscle mass, using estimated renal function alone may not be appropriate for dose adjustment. In this case, the creatinine clearance calculated from the serum and 24-h urine collection on POD 30 was 39 mL/min, whereas the creatinine clearance derived from the serum creatinine level and the Cockcroft–Gault equation was 51 mL/min. Additionally, the eGFR based on the serum cystatin C level measured on POD 246 was 31.5 mL/min/1.73 m^2^, which was lower than the eGFR of 50.9 mL/min/1.73 m^2^ calculated from serum creatinine at the same time. These results suggest that the actual renal function of this patient might have been lower than that estimated using the serum creatinine levels. As mentioned above, ganciclovir is primarily excreted in urine, and its clearance correlates with GFR [12]. Therefore, overestimation of creatinine clearance could result in excessive exposure to ganciclovir, potentially leading to an overdose. Taken together, the TDM of ganciclovir may be beneficial in situations where accurate prediction of renal function is challenging.

In the present case, the valganciclovir dosage was adjusted to maintain the ganciclovir trough concentration within the range of 300–800 ng/mL, with no observed CMV reactivation. Few consensuses on the TDM range for prophylaxis have been established. The lower limit mirrors the efficacy of ganciclovir prophylaxis. While drug-resistant CMV infection is uncommon post-lung transplantation, patients who develop it are noted to have reduced overall survival [13]. In this case, only trough concentrations of ganciclovir were monitored. However, few studies assessed the relationship between trough concentration and the area under concentration–time curve (AUC) of ganciclovir, and a wide range of correlation coefficients between trough concentrations and AUC has been observed [14]. Hence, further prospective pharmacokinetic studies are required to determine the optimal concentration for prophylaxis.

The initiation of valganciclovir treatment led to grade 2 leukopenia and grade 1 neutropenia in this case. Nonetheless, platelet counts were not significantly affected by valganciclovir administration. Similar outcomes were observed in a prior study involving solid organ transplant recipients receiving oral valganciclovir or ganciclovir, where ganciclovir exposure correlated with leukopenia and neutropenia but not thrombocytopenia [15]. These findings suggest that higher blood concentrations of ganciclovir are likely to worsen neutropenia and leukopenia, among other hematologic toxicities. Nevertheless, there are reports that no correlation exists between ganciclovir blood levels and hematologic toxicity [16, 17], possibly owing to differences in concomitant drugs among patients in these studies. Future research should focus on clarifying the effects of ganciclovir or valganciclovir on leukopenia and neutropenia, particularly regarding drug interactions or the influence of concomitant drugs.

This case report suggests that the dosage of valganciclovir should be adjusted based on trough serum ganciclovir concentration in patients receiving lung transplants. TDM of ganciclovir is a useful tool for clinicians to achieve successful prophylaxis with valganciclovir with tolerable safety.

## Data Availability

Data used in this case report will not be shared owing to the potential risk of identifying the individual.

## Abbreviations

CMV: Cytomegalovirus
TDM: Therapeutic drug monitoring
POD: Postoperative day
eGFR: Estimated glomerular filtration rate
AUC: Area under concentration–time curve
